# How High-Polluting Firms Suffer from Being Distracted form Intended Purpose: A Corporate Social Responsibility Perspective

**DOI:** 10.3390/ijerph17249197

**Published:** 2020-12-09

**Authors:** Xue-Zhou Zhao, Jun Chen, Feng-Wen Chen, Wei Wang, Senmao Xia

**Affiliations:** 1 School of Management and Engineering, Nanjing University, Nanjing 210093, China; xzhao@nju.edu.cn (X.-Z.Z.); chenjun@smail.nju.edu.cn (J.C.); wwqd2hs@smail.nju.edu.cn (W.W.); 2 School of Economics and Business Administration, Chongqing University, Chongqing 400030, China; 3 Business School, Coventry University, Coventry CV15FB, UK; Senmao.Xia@coventry.ac.uk

**Keywords:** corporate innovation, corporate social responsibility, financialization, high-polluting industries, environmental protection

## Abstract

High-polluting industries are regarded as the main sources of air pollutant emissions and the major factors that significantly destroy the ecological environment. Corporate innovation in high-polluting industries improves the energy consumption efficiency and reduces the emission of air pollutant, which mitigates the conflict between environment and economy. Using the sample of China’s listed firms from 2010 to 2017, this study examines the impact of corporate social responsibility (CSR) and financialization on corporate innovation in high-polluting industries. The results show that there is a positive association between CSR and corporate innovation, while there is a negative association between financialization and corporate innovation. Furthermore, the financialization of high-polluting firms can alleviate the promotion role of CSR in the innovation process. The financialization of state-owned enterprises in high-polluting industries may not have a crowding-out effect on research and development (R&D), but it can limit the R&D promotion effect of CSR engagements. In contrast, the financialization of non-state-owned enterprises will hinder corporate innovation, but it will not affect the association between CSR and technology innovation. We also find that the financialization of high-polluting firms with low financial constraints can alleviate the promotion role of CSR engagements in innovation. Meanwhile, the CSR engagements of high-polluting firms with high financial constraints play a stronger role in corporate innovation. During the implementation of environmental policies, the negative association between financialization and corporate innovation has been strengthened. Our findings can encourage high-polluting firms to make more efforts in environmental protection and social stability.

## 1. Introduction

In recent decades, air pollutant has been regarded as a serious environmental pollution, and it is also a great threat to the environment of human being. With the decline of air quality, more and more diseases produce many problems to governments [1]. Faced with this challenge, international society have formulated many environmental policies for energy-saving and emission-reduction, such as The Paris Agreement in 2015 [2,3]. The purpose of these environmental policies is to reduce the pollutant emissions of manufacturing firms, and keep the rise in temperature below 2 °C [4]. Although environmental policies may relieve the impact of air pollution on the environment, in developing countries, there are also some realistic factors to hinder the government’s implementation of environment-friendly policies, such as the relocation of manufacturing firms from developed countries to the developing countries. High-polluting firms, the main producers of air pollutants, is also one of the key drivers of economic growth in developing countries. As a result, high-polluting firms provide researchers and governments with a dilemma: whether we should protect the environment with restricting the production of high-polluting firms, or encourage economic growth by sacrificing the environment [5]. In order to accelerate economies, most developing countries have attempt to accept the approach of treatment after pollution [6]. Although China is still a developing country, with its achievements in economic development, this dilemma is more apparent. As a result, China does not only implement more strict environmental policies, such as The Environmental Protection Law of China in 2014, but also encourages firms to be more proactive in environment protection [2,3].

For example, China encourages high-polluting firms to improve the resource consumption efficiency and reduce the emission of air pollutant. Thus these firm’s investment in corporate innovation could be viewed as an important role in resolving this conflict between environment and economy. Corporate innovation can improve the profitability of firms, and also drive technological upgrading [7]. In addition, corporate innovation can effectively mitigate environmental damages caused by the pollutant emissions of high-polluting firms, and then support environmental protection [8]. However, there are two factors that hinder the corporate innovation in high-polluting firms. First, innovation is resource consuming, and firms must consider their business strategies before the decision-making of innovation [9]. Faced with resource constraints, high-polluting firms can only invest limited resources in research and development (R&D) activities for achieving environmental benefits. Second, it is difficult to estimate the economic benefits and the beneficiaries of corporate innovation in the short run [10]. Therefore, corporate innovation may be affected by the diversified needs of stakeholders and the interests of shareholders.

**Figure 1:**
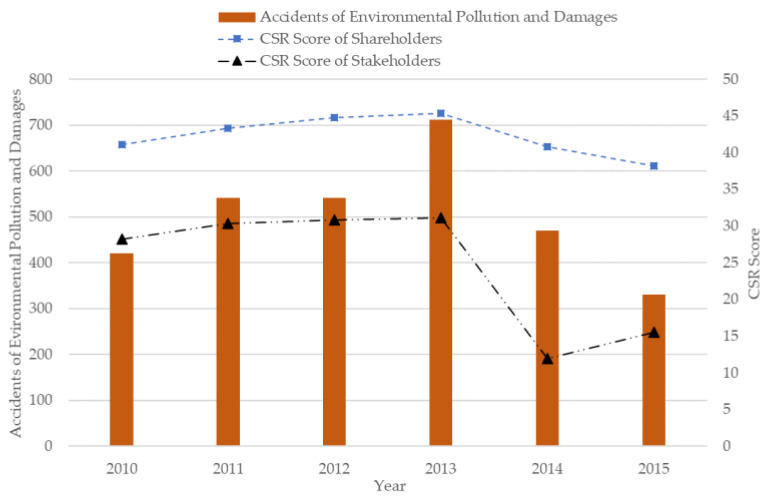
Environmental pollution and corporate social responsibility (CSR) in China.

One way to reconcile the diversified needs is corporate social responsibility (CSR), which has restricted the managers’ only desire for profit maximization. CSR requires firms to make contributions to society and environment [11]. Although CSR could be as a continuum of possibilities going from serious environmental commitment to the facelift changes generally referred to as “greenwashing”, Lee, Cruz, and Shankar (2018) also show that allowing “greenwashing” may incentivize some firms to go genuinely green as long as there are some informed customers in the market [12]. So in general we still believe that CSR can effectively be translated into effective wide-ranging policy changes. For high-polluting firms, CSR engagement provides specific information about the efforts in the social stability, and serves as a signal for more recognition from shareholders and the other stakeholders. However, some CSR engagements of manufacturing firms can be regarded as business strategy, and put more focus on their shareholders. In Figure 1, it can be seen that the changes of shareholders-related activities in high-polluting industries, which obtained from the China Stock Market and Accounting Research (CSMAR) Database, better match the accidents of environmental pollution and damages in China from 2010 to 2015. It is interesting that stakeholders-related activities in high-polluting industries was at a surprisingly low point in 2014, and this might be caused by the promulgation of The Environmental Protection Law [3]. For high-polluting industries, managers pay more attention to profit maximization and resource exploitation, and this can trigger an argument that whether there is a close association between CSR and corporate innovation [13].

**Figure 2:**
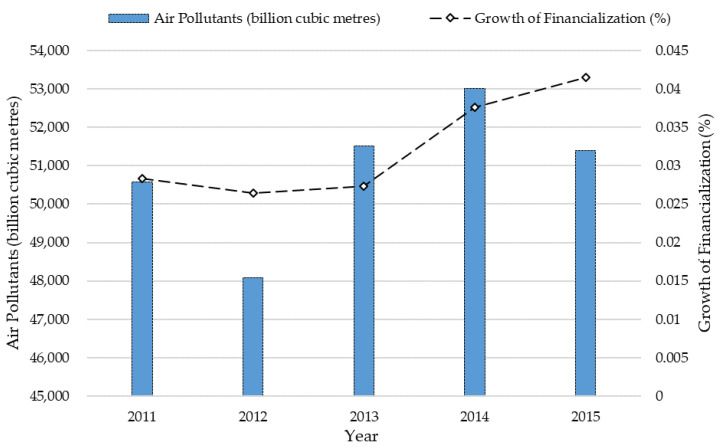
Air pollutants and financialization of high-polluting industries in China.

One way to meet the demands of shareholders in high-polluting firms is to invest in finance and real estate, which is called the financialization. Compared with product business, financialization can bring higher profits, but be also accompanied by higher risks. As non-financial industries, high-polluting industries are very special in operations, and their productions are also strictly controlled by governments. For this reason, high-polluting firms tend to choose the strategy of financialization to improve their profitability. It is worth noting that financialization can change the asset structure of high-polluting firms, and also reduce some investments in corporate innovation [14], and then influence the efforts in the environment protection. As shown in Figure 2, the growth of average financialization in high-polluting industries are consistent with the emission of air pollutants from 2011 to 2015, indicating that more financial asserts may cause more environmental damages. Financialization of high-polluting firms might be an important influencing factor on the unsatisfied air quality [15]. Financialization is a serious impediment to corporate innovation, and meanwhile, it helps distinguish shareholder-oriented and stakeholder-oriented CSR engagements. Exploring the impact of financialization on CSR can help high-polluting firms to obtain the optimal decision of innovation, and establish the relationship between stakeholders and shareholders.

Empirically, this study explores the impact of CSR and financialization on corporate innovation based on a sample of China listed companies in high-polluting industries from 2010 to 2017. The empirical results show that CSR can promote corporate innovation, while financialization can inhibit corporate innovation. Moreover, the financialization of high-polluting firms can change the role of CSR in promoting innovation, especially in related activities. In the Chinese market, the financial assets of high-polluting firms can influence stakeholders’ asset structure, and financialization will also change the dominant position of CSR in promoting innovation. Considering different ownerships, the CSR engagements of state-owned enterprises can promote corporate innovation, while there are not significant association between financialization and corporate innovation. The shareholders-related activities of non-state-owned enterprises can promote corporate innovation, while financialization can hinder their innovation outcomes. It is worth noting that the financialization of state-owned enterprises can only alleviate the positive associations between stakeholders-related activities and corporate innovation. In terms of financial conditions, CSR can promote corporate innovation under different financial constraints, while financialization can only inhibit corporate innovation under the condition of high financial constraints. Specifically, the financialization of high-polluting firms can alleviate the association between CSR and corporate innovation under the condition of low financial constraints, but high financial constraints will allow managers to pay more attention to the high profits of financialization. After the implementation of environmental policy, the impact of financialization on corporate innovation is strengthened, and it can alleviate the dominant position of stakeholders-related activities in promoting innovation outcomes.

This study provides a better understanding of the association among CSR, financialization, and corporate innovation. The main contributions are as follows: First, based on signal theory, this study demonstrates a positive association between CSR and corporate innovation, so high-polluting firms need to transfer the corporate practices of social responsibility to environmental benefits through innovation outcomes. Second, the association between financialization and corporate innovation reveals that the financialization will influence the quantity and quality of innovation outcomes. Finally, the performance gap between stakeholders-related and shareholders-related activities demonstrates that high-polluting firms may use CSR engagements to achieve the goal of profit maximization, and the “shift from real to virtual” of high-polluting firms in the Chinese market can reduce the environmental benefits of technology upgrades.

The structure of this study is as follows: Section 2 provides the literature review and proposes the research hypotheses. Section 3 describes the research design. Section 4 presents the empirical results. Section 5 discusses the findings from empirical results. Section 6 provides the conclusions and recommendations.

## 2. Literature Review and Hypotheses

### 2.1. Corporate Social Responsibility and Corporate Innovation

Corporate social responsibility reflects the contribution of firms to society, environment, customers, suppliers, and employees, and is also an important factor to measure the sustainable ability of firms. For high-polluting industries, managers often suffer from some problems of environmental damages, and must take into account the expectations of the public [6]. As the operation of high-polluting firms is a major source of environmental pollution, the technological improvement of such firms is more important than that of other manufacturing firms. Signaling theory refers to the theory first proposed by Spence (1973), who won the Nobel Prize in economics in 2001. If market participants have asymmetric information, “signal” can provide useful information for decision-making. For example, a signal from debt management is that the company has good expectations for future earnings. Famous brand goods convey an accurate signal to consumers: it is a kind of high-quality products, which should be more expensive and more valuable than ordinary commodities. This theory can also explain why enterprises prefer to pay dividends to employees rather than cash. From the perspective of signal theory, dividend payment strongly expresses the company’s good prospects. According to signaling theory, high-polluting firms need to use CSR engagements to deliver some information about their non-opportunistic behavior to meet the expectations of the society [16]. These special signals can improve the reputation of high-polluting firms, and help them to gain legitimacy [17].

From the perspective of stakeholder and legitimacy, many empirical studies have shown that there is a positive association between CSR and corporate innovation. Cegarra-Navarro et al. found that companies disclose innovation outcomes to reveal their social responsibility practices [11]. Wu et al. got a positive correlation between CSR and corporate innovation, and pointed that this relationship can be also mediated by the visibility and transparency of firms [18]. Briones et al. got a similar result that there is a positive correlation between CSR and corporate innovation [19]. Hu et al. pointed out that CSR is a way to deliver some information about firms’ operations [20]. Shahzad et al. made an analysis of the association between CSR and environmental sustainability, and found that social responsibility practices can significantly contribute to the green innovation of firms [5]. Wu et al. found that firms with higher scores of CSR will pay more attention to their pollutant emissions [8]. Jiang et al. pointed out that environmental disclosure can have a significant positive impact on corporate innovation [21]. From the perspective of external financing, CSR is an important factor in improving corporate performance. Bocquet et al. pointed out that the contribution of CSR to corporate performance is primarily driven by corporate innovation [22]. Martinez-Conesa et al. found that both of CSR and innovation performance can contribute to corporate performance, and CSR is the main driver of corporate innovation [23].

Based on signaling theory and stakeholder theory, high-polluting firms need to rely on more innovation outcomes to express their CSR engagements [8]. This is because more corporate practices in environmental protection will get more attention of stakeholder, so that their investments in CSR engagements can meet the expectations of the society. Through delivering some information about CSR, high-polluting firms will protect the interests of stakeholders, and also gain legitimacy. Therefore, this study proposes the following hypothesis:

**Hypothesis** **1** **(H1).** *There is a positive association between CSR and corporate innovation in high-polluting industries.*

### 2.2. Financialization and Corporate Innovation

Financialization represents the increasing proportion of investment in financial assets, which leads to more and more operating profits of non-financial firms from financial channels rather than manufacturing stages. The intention of financialization is to improve the profit structure of non-financial firms, so that the interests of shareholders can be protected. In the Chinese market, more and more manufacturing firms have begun to “shift from real to virtual” [14]. However, as a high knowledge-intensive activity, corporate innovation needs firms to allocate internal resources reasonably. Moreover, the sunk cost of innovation outcomes is relatively great, so that firms need to be cautious before the decision-making of corporate innovation. Therefore, the relationship between financialization and corporate innovation mainly reflects the future development strategy of firms.

For the financialization of non-financial firms, the main characteristics, influence factors, and economic consequences of financialization have been discussed in many finance studies. Bloom et al. found financialization can mitigate the impact of external uncertainty on corporate development, in order to help firms avoid some economic risks [24]. Soener pointed out that the financialization of non-financial firms can improve the efficiency of capital operation, and reduce their financial constraints [25]. Izhar Baranes found that financialization can help some firms to improve the accumulation of intangible assets, thus expanding their income channels [26]. Barradas and Lagoa made an analysis of the association between financialization and investment behavior of non-financial firms, and found that financialization can hinder the real investment of such firms by weakening their long-term profitability [27]. Bowman pointed out that financialization may be mainly formed by the pressure of shareholders, and this can lead to the unbalanced distribution of capital resources within firms [15]. Moreover, financialization can inhibit the real investment of firms, and create a decline in their productivity [14]. Zhou et al. found similar findings, and pointed out that the financialization of non-financial firms can reduce their productivity [28]. Pang and Wang found that financialization can reduce non-financial firms’ investment in R&D, and this is the main reason for the decrease in production [29].

Financialization has a crowding out effect on the investment in R&D. In other words, if non-financial firms invest more and more in finance and real estate, these firms can only reduce the investment in R&D, thus inhibiting corporate innovation. Although some short-term profits from the financialization can meet the demands of shareholders, it will seriously destroy the capital structure of non-financial firms. In this situation, the internal resources for R&D process may be insufficient, and then the quality and quantity of innovation outcomes cannot be guaranteed. Therefore, this study proposes the following hypothesis:

**Hypothesis** **2** **(H2).** *There is a negative association between financialization and corporate innovation in high-polluting industries.*

### 2.3. Corporate Social Responsibility and Financialization

For high-polluting industries, the association between CSR and corporate innovation may be influenced by financialization. From the perspective of resource constraints, CSR engagements can inhibit corporate innovation under the shortage of innovation resources. In the Chinese market, high-polluting firms often focus on their images and profits rather than sustainable abilities [30]. In order to maximize the profits of shareholders, high-polluting firms may regard some CSR engagements as a special business strategy, which can also promote corporate performance [31]. Li and Wu pointed that there is a performance gap of CSR engagements between stakeholders-related and shareholders-related activities [13]. The findings of Cupertino et al. also found this performance gap, indicating that financialization can promote the capital accumulation of firms with some social responsibility practices [32]. Lu et al. made an analysis of the impact of different dimensions of CSR on corporate competitiveness, and found that the environmental and social dimensions of CSR can be the main factors in promoting financial performance [33]. Lin et al. found that CSR has a positive impact on financial performance [34]. However, high-polluting firms prefer choosing the way of financialization for meeting the demands of shareholders [35]. In this situation, high-polluting firms will invest more and more into the financial and real estate industries, and pay more attention to the economic benefits of CSR engagements, which may boost the shortage of innovation resources. Therefore, this study proposes the following hypothesis:

**Hypothesis** **3** **(H3).** *The positive association between CSR and corporate innovation is alleviated by the financialization of high-polluting firms.*

State-owned enterprises have more advantages in policies and assets. First, state-owned enterprises show higher levels of CSR engagements, compared to firms with other ultimate owners. For example, Lopatta et al. (2017) found a positive relation between state-controlled ownership and the CSR performance of firms worldwide [36]. They argued that the governments, as the controlling shareholders of the firm, always have incentives to pursue social stability, and they are capable to achieve CSR goals: they appoint executive managers, and have more resources to implement CSR plans. Second, state-owned and non-state-owned high-polluting firms may show different levels of green innovation. In recent years, Chinese government has made continuous efforts in environmental protection, and state-owned enterprises are the vanguard. According to the List of China’s Top 100 Green Gold Enterprises in 2018, the state-owned enterprises have the largest number of shortlisted enterprises [37]. It is the social expectation for the state-owned enterprises to take the lead in green development. One reason is that state-owned enterprises can receive a much higher proportion of government subsidies than those of non-state-owned enterprises [33]. Another reason may be that managers from state-owned high-polluting firms need to show innovation outcomes to express their contributions to the society and environment in order to accumulate political capital. On the other hand, because the positive spillover of innovation will reduce the imitation cost of competitors, non-state-owned enterprises need to disclose the information about corporate innovation selectively. Third, literature that links CSR and innovation shows in developing countries, only firms with government support could exhibit the positive CSR effect on innovation [38]. So, we expect to find difference on the association between CSR and innovation for state-owned and non-state-owned enterprises. Finally, we expect the crowding out effect of financialization on innovation may vary considerably across the different ultimate ownership groups. Because of the abundant resources of state-owned enterprises, their innovation activities can be supported by internal resources, which alleviates the financialization’s crowding out effect.

**Hypothesis** **4a** **(H4a).** *The financialization of state-owned enterprises in high-polluting industries does not have a negative impact on corporate innovation, but it alleviates the positive association between CSR and corporate innovation.*

**Hypothesis** **4b** **(H4b).** *The financialization of non-state-owned in high-polluting industries has a negative impact on corporate innovation, but it does not alleviate the positive association between CSR and corporate innovation.*

Because of information asymmetry and policy constraints, the cost of internal financing is much lower than that of external financing. Corporate innovation in high-polluting industries suffers from high risks and continuous investments [39]. When the financial constraints of high-polluting firms are at a low level, their sufficient funds can support the activities of corporate innovation. Cheng et al. found that CSR can lower the external financial constraints from the social and environmental dimension [40]. Ruggiero and Cupertino analyzed the association between CSR and financial performance, and found that more social responsibility practices will bring more financing opportunities for firms [41]. When firms face high financial constraints, financialization will take up most of their internal resources, so that it is more difficult to support the activities of R&D [42]. Therefore, this study proposes the following hypothesis:

**Hypothesis** **5a** **(H5a).** *Faced with low financing constraints, the financialization of high-polluting firms does not have a negative impact on corporate innovation, but it alleviates the positive association between CSR and corporate innovation.*

**Hypothesis** **5b** **(H5b).** *Faced with high financing constraints, the financialization of high-polluting firms has a negative impact on corporate innovation, but it does not alleviate the positive association between CSR and corporate innovation.*

The decision-making of high-polluting firms is often restricted by environmental policies, including energy consumption, product production, and pollutant emission. Therefore, high-polluting firms should make more efforts in the environmental and social dimension, and also attach importance to the potential risks of capital operation. Chan et al. found that the pressures of environmental policy can promote companies to invest more in green innovation [43]. Wang and Yuan pointed out that the control intensity of pollutant emissions can drive the technological innovation of high-polluting firms [44]. Qin et al. believed that China’s green transition can promote high-polluting firms to invest more and more in green innovation [45]. In addition, environmental policies will also have an impact on the capital structure of high-polluting firms, so that such firms need to use some investments with high profits to meet the needs of shareholders. Rodrigues et al. analyzed the impact of environmental policy on the decision-making of corporate innovation, and pointed out that environmental policy can change the capital structure of non-financial firms [46]. Under the control of macro policies, the cash flow and profitability of high-polluting firms can be influenced by these environmental policies [47]. Therefore, this study proposes the following hypothesis:

**Hypothesis** **6a** **(H6a).** *Before the implementation of environment policy, the financialization of high-polluting firms does not alleviate the positive association between CSR and corporate innovation.*

**Hypothesis** **6b** **(H6b).** *After the implementation of environmental policy, the financialization of high-polluting firms alleviates the positive association between CSR and corporate innovation.*

**Figure 3:**
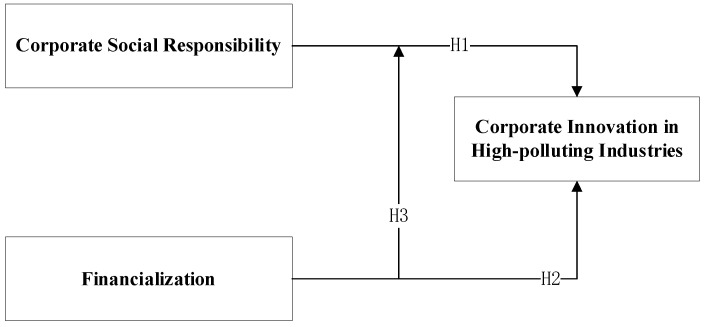
Research model.

The research model presented in this study is presented in Figure 3.

## 3. Research Design

### 3.1. Data Sources

The research sample initially is comprised of China’s A-share listed firms on the Shenzhen Stock Exchange and the Shanghai Stock Exchange from 2010 to 2017. This study chooses 2010 as the beginning year of sample period, because the Ministry of Ecology and Environment of People’s Republic of China published the “Guidelines on Environmental Information Disclosure of Listed Companies” in 2010, which defined the high-polluting industries in detail. This environmental policy expanded the kinds of high-polluting industries from 14 to 16, according to the “Management List of Listed Companies in Environmental Protection Verification Industries” released in 2008. New high-polluting industries include thermal power, steel, cement, electrolytic aluminum, coal, metallurgy, chemical, petrochemical, building materials, paper, brewing, pharmaceutical, fermentation, textile, leather, and mining. Therefore, this study selects 16 high-polluting industries from the listed companies of Shenzhen Stock Exchange and Shanghai Stock Exchange as the research sample. In order to ensure the reliability of data, this study excludes sample firms with incomplete data and deletes the delisted companies. Finally, 3428 observations of high-polluting industries in the Chinese market are obtained.

The main data sources of this study are the Hexun CSR Database, the China Stock Market and Accounting Research (CSMAR) Database. The evaluation data of CSR are obtained from the Hexun CSR Database, and its CSR score is the most widely used to measure the CSR evaluation of China’s listed companies [48]. The data of corporate innovation come from the patent database of CSMAR Database, and the data of financialization is derived from the financial statement database of CSMAR Database. For control variables, the data of corporate finance and governance are derived from the CSMAR Database. All continuous variables are winsorized at 1% at both tails, which can minimize the influence of extreme values in empirical analysis.

### 3.2. Variables

#### 3.2.1. Dependent Variable

Corporate innovation (Innovation) represents the innovation capability and outcome of non-financial firms. In high-polluting industries, corporate innovation is often demonstrated through technological innovation outcomes, which can improve the energy consumption and reduce the pollutant emissions for high-polluting firms. Referring to Chen et al. this study uses the natural logarithm of patent applications plus one to measure the corporate innovation in high-polluting industries [7].

#### 3.2.2. Independent Variables

Corporate social responsibility (CSR) represents the contribution of high-polluting firms to environment, society, and consumers, and it is an important indicator of efforts made by these firms in environmental protection. In the Chinese market, the CSR of high-polluting firms should be combined with the characteristics of industry and market [48]. The Hexun Database evaluates CSR from five dimensions, including shareholders, employees, suppliers, environment, and society. The characteristics of the Chinese market are fully considered in the CSR evaluation system of Hexun, which provides the objective evaluation of CSR engagements in high-polluting industries. Therefore, this study uses the total score of CSR to measure the CSR evaluation of high-polluting firms.

Financialization represents the proportion of firms’ investments in financial assets, which can produce more operating profits from financial and real estate industries. The financialization of high-polluting firms can occupy large internal resources, which causes managers to pay more attention to this short-term profit from financial assets. Referring to Tori and Onaran, this study measures the financialization of high-polluting firms by using the ratio of financial assets to total assets [14]. Financial assets include derivative financial instruments, trading financial assets, net available for sale financial assets, net long-term investments on bonds, net hold-to-maturity investments, net short-term investments, disbursement of loans and advances, long-term financial equity investments, net investment properties, and entrusted investments and trust products in other liquid assets.

#### 3.2.3. Control Variables

Following prior studies, this study selects control variables from organizational characteristics, corporate finance, and corporate governance which can affect the corporate innovation [49,50]. The control variables include return on assets, leverage, company, company, asset turnover, Tobin Q, current assets, company growth, proportion of independent directors, and shareholding ratio of top three shareholding. Return on assets (ROA) measures the profitability of company and is calculated as the net income derived by the total assets. Leverage is calculated as the total debts divided by total assets. Company age is measured by the duration from the year in which the firm was established to the sample year. Asset turnover is calculated as the total revenge divided by total assets. Tobin Q is calculated as the market value divided by total assets. Current assets (Liquidity) is calculated as the current assets divided by the total assets. Company growth (Sales) is calculated as the growth rate of operation revenues. Proportion of independent directors represents the governance structure and is calculated as the proportion of independent directors on the board of directors. Shareholding ratio of top three shareholders (Hold) represents the shareholding structure of high-polluting firms. Table 1 provides the definitions of all variables used in empirical analysis.

### 3.3. Models

In order to test the hypothesis proposed in Section 2, this study uses panel data models to analyze the association among CSR, financialization, and corporate innovation. Considering the influence of potential endogeneity, independent variables and control variables lag one year and the dependent variable (Innovation) is in its current value. The empirical models also control the year and the industry fixed effects. The basic empirical model is as follows:

(1) $$\begin{array}{ll} Innovation_{i,t}= & \alpha_{0}+\beta_{1}CSR_{i,t-1}+\beta_{2}FIN_{i,t-1}+\beta_{3}ROA_{i,t-1}+\beta_{4}Leverage_{i,t-1}\\ & +\beta_{5}Age_{i,t-1}+\beta_{6}Asset\;turnover_{i,t-1}+\beta_{7}Tobin\;Q_{i,t-1}\\ & +\beta_{8}Liquidity_{i,t-1}+\beta_{9}Sales_{i,t-1}+\beta_{10}Independ_{i,t-1}+\beta_{11}Hold_{i,t-1}\\ & +Year_{fixed\;effects}+Industry_{fixed\;effects}+\varepsilon \end{array}$$

In Equation (1), $Innovation_{i,t}$ represents the corporate innovation of firm *i* in year *t*. $CSR_{i,t-1}$ represents the CSR score of firm *i* in year *t* − 1. $FIN_{i,t-1}$ represents the financialization of firm *i* in year *t* − 1. $ROA_{i,t-1}$ represents the profitability of firm *i* in year *t* − 1. $Leverage_{i,t-1}$ represents the debts of firm *i* in year *t* − 1. $Age_{i,t-1}$ represents the company age of firm *i* in year *t* − 1. $Asset\;turnover_{i,t-1}$ is defined as the ratio of sales revenue to average total assets of firm *i* in year *t* − 1. $Tobin\;Q_{i,t-1}$ represents the market value of firm *i* in year *t* − 1. $Liquidity_{i,t-1}$ represents the current assets of firm *i* in year *t* − 1. $Sales_{i,t-1}$ represents the increased percentage of sales revenue for firm *i* in year *t* − 1. $Independ_{i,t-1}$ represents the proportion of independent directors for firm *i* in year *t* − 1. $Hold_{i,t-1}$ represents the shareholding ratio of top three shareholders for firm *i* in year *t* − 1. $Year_{fixed\;effects}$ can control the year fixed effects, and $Industry_{fixed\;effects}$ can control the industry fixed effects. ε is the error term.

(2) $$\begin{array}{ll} Innovation_{i,t}= & \alpha_{0}+\beta_{1}CSR_{i,t-1}+\beta_{2}FIN_{i,t-1}+\beta_{3}CSR_{i,t-1}\times FIN_{i,t-1}+\beta_{4}ROA_{i,t-1}\\ & +\beta_{5}Leverage_{i,t-1}+\beta_{6}Age_{i,t-1}+\beta_{7}Asset\;turnover_{i,t-1}\\ & +\beta_{8}Tobin\;Q_{i,t-1}+\beta_{9}Liquidity_{i,t-1}+\beta_{10}Sales_{i,t-1}\\ & +\beta_{11}Independ_{i,t-1}+\beta_{12}Hold_{i,t-1}+Year_{fixed\;effects}\\ & +Industry_{fixed\;effects}+\varepsilon \end{array}$$

To further examine the moderating effect of financialization on the association between CSR and corporate innovation, the interaction of CSR and FIN ($CSR_{i,t-1}\times FIN_{i,t-1}$) is introduced into the basic model of Equation (1) to construct Equation (2). Equation (2) will demonstrate the role of financialization in the innovation decision of high-polluting firms.

## 4. Empirical Results

### 4.1. Descriptive Statistical Analysis

Table 2 presents the descriptive statistics of all variables used in empirical models. The mean and standard deviation of Innovation are 2.1725 and 1.7566 respectively, indicating that there is a big gap in patent applications among high-polluting firms. The mean and median of CSR are 28.8457 and 22.3500 respectively, indicating that most high-polluting firms are not good at social responsibility practices. In addition, the standard deviation and maximum of CSR are 21.0923 and 78.2600 respectively, which demonstrates that there are some differences in CSR engagements among high-polluting firms. For the financialization of high-polluting firms, the difference between its mean (0.0357) and median (0.0006) is relatively large, demonstrating that a few high-polluting firms rely too heavily on financial assets. When coming to the control variables, the minimum of ROA is −0.1768, which indicates that some high pollution enterprises are in the red. The maximum and mean of Leverage are 0.9934 and 0.5066 respectively, indicating that the operations of the most high-polluting firms are supported by their debts. For Asset turnover, its mean and maximum are 0.7028 and 2.4732 respectively, which demonstrates that there are great differences in total assets and total revenue among high-polluting firms. The results of Tobin Q show that there is a big gap in terms of market value. The mean and standard deviation of Liquidity are 0.4361 and 0.1973 respectively, which indicates that the distribution of current assets in high-polluting industries is balanced. The maximum of Hold is 0.7056, demonstrating that there is a problem of over-centralization of shares in some high-polluting firms.

This study further analyzes the correlation of all variables, and the results of Pearson correlation Matrix are reported in Table 3. The correlation coefficient between CSR and Innovation is 0.175, significant at the 1% level, indicating that there is a positive association between CSR and corporate innovation. The correlation coefficient between FIN and Innovation is −0.124, significant at the 1% level, demonstrating that there is a negative association between financialization and corporate innovation. The results of CSR or FIN can be used to support the hypotheses in Section 2. The absolute correlation coefficients between all control variables and Innovation are less than 0.5, which indicates that independent variables and control variables can effectively explain corporate innovation. The VIF test is used to analyze the problem of multi-collinearity, and the VIF values of all variables are less than 2, demonstrating that the empirical models are not affected by multi-collinearity.

### 4.2. Regression Results

#### 4.2.1. Baseline Results

In order to explore the impact of CSR and financialization on corporate innovation, this study uses the empirical models based on Equation (1) and Equation (2) to test the hypotheses proposed in Section 2. First of all, the univariate analysis is used to explore the impact of CSR or financialization on corporate innovation in high-polluting industries. Then, control variables are introduced into the process of regression analysis. Finally, the interaction between CSR and financialization is introduced into empirical analysis. The baseline results of overall samples are reported in Table 4.

In Table 4, Columns (1) and (2) respectively explore the impact of CSR and financialization on corporate innovation without considering the control variables. The coefficients of CSR and financialization are 0.0187 and −2.2325, both significant at the 1% level. After introducing control variables, the results of Columns (3) and (4) indicate that there is a significant positive association between CSR and corporate innovation, while there is a significant negative association between financialization and corporate innovation. Column (5) explores the impact of CSR and financialization on corporate innovation, and the coefficients of CSR and financialization are 0.0164 and −1.5928, both significant at the 1% level. The results of Columns (1)–(5) demonstrate that CSR can promote corporate innovation in high-polluting industries, while financialization can inhibit corporate innovation in such industries. These results can support H1 and H2 proposed in Section 2. The moderating effect of financialization is explored in Column (6). The coefficient of interaction term (CSR*FIN) is −0.0497, significant at the 5% level, indicating that the financialization of high-polluting firms can alleviate the positive association between CSR and corporate innovation, which supports H3. The findings suggest that the financialization of high-polluting firms can achieve the goal of profit maximization for meeting the demands of shareholders in a short time, which makes managers to invest more and more in financial assets, thus reducing the R&D expenditures.

#### 4.2.2. Regression Results of Different Ownership

The production of high-polluting firms should be supported by external resources. In the Chinese market, state-owned enterprises in high-polluting industries own more advantages in assets and policies than the non-state-owned enterprises [51]. For this reason, this study divides the research sample of high-polluting industries into the subsample of state-owned enterprises (SOEs) and the subsample of non-state-owned enterprises (Non-SOEs). The regression results of different ownership are reported in Table 5.

In Table 5, Columns (1) and (2) respectively explore the impact of CSR and financialization on corporate innovation in the subsample of SOEs. The coefficient of CSR is 0.0145, significant at the 1% level. In the results of Column (3), there is a positive association between CSR and corporate innovation, while the impact of financialization is not significant. Column (4) explores the moderating effect of financialization, and the coefficient of the interaction term (CSR*FIN) is −0.0731, significant at the 5% level. The results of Column (4) indicate that the financialization of SOEs can alleviate the impact of CSR on promoting innovation, which supports H4a. Column (5) and (6) respectively explore the impact of CSR and financialization on corporate innovation in the subsample of Non-SOEs. The coefficients of CSR and financialization are 0.0074 and −2.3975, respectively significant at the 5% level and 1% level. In the results of Column (7), there is a positive association between CSR and corporate innovation, while there is a negative association between financialization and corporate innovation. The moderating effect of the financialization in Non-SOEs is explored in Column (8). The results of Column (8) show that the financialization of Non-SOEs cannot inhibit the association between CSR and corporate innovation, which supports H4b. In different subsamples, CSR can promote corporate innovation in high-polluting industries, while financialization can only inhibit the innovation outcomes of Non-SOEs. These findings suggest that the financialization of SOEs can alleviate managers’ innovation intention through CSR engagements. Furthermore, the financialization of Non-SOEs can reduce their R&D expenditures, but it cannot influence the managers’ motivations for technology upgrades in the dimension of environment and society.

#### 4.2.3. Regression Results of Financial Constraint

Under different financial constraints, high-polluting firms mainly rely on internal financing to support R&D activities. Referring to Hadlock and Pierce, this study calculates the levels of financial constraints faced by high-polluting firms in the Chinese market [52]. The research sample is divided into two subsamples: the subsamples with low financial constraints (financial constraints are higher than the median) and the subsamples with high financial constraints (financial constraints are lower than the median). The regression results of different financing constraints are reported in Table 6.

In Table 6, Columns (1) and (2) respectively explore the impact of CSR and financialization on corporate innovation under low financial constraints. The coefficient of CSR is 0.0179, significant at the 1% level, while financialization has no significant effect on corporate innovation. In the results of Column (3), CSR can promote the innovation outcomes of high-polluting firms. Column (4) explores the moderating effect of financialization under the condition of low financial constraints, demonstrating that financialization can alleviate the impact of CSR on innovation, which supports H6a. Columns (5) and (6) respectively explore the impact of CSR and financialization on corporate innovation under the condition of high financial constraints. The coefficients of CSR and financialization are 0.0096 and −2.1601, both significant at the 1% level. In the results of Column (7), there is a positive association between CSR and corporate innovation, while there is a negative association between financialization and corporate innovation. Column (8) explores the moderating effect of financialization with high financing constraints, and its results indicate that financialization cannot alleviate the association between CSR and corporate innovation, which supports H6b. These findings suggest that the financialization of high-polluting firms with low financial constraints will not have a crowding out effect on R&D expenditures, but it can influence managers’ innovation intention.

#### 4.2.4. Regression Results of Environmental Policy

Considering the association between high-polluting industries and environmental pollutants, the operation and production of high-polluting firms will be limited by environmental policies. Environmental policies will have a direct impact on the energy consumption and pollutant emission of high-polluting firms, and encourage such firms to make more contributions to environmental protection [3]. Ministry of Ecology and Environment of People’s Republic of China improved The Environmental Protection Law in 2014, and required non-financial firms to implement more activities for environmental protection. Therefore, this study divides the research sample into the subsamples before 2014 and the subsamples after 2014. The regression results of different periods are reported in Table 7.

In Table 7, Columns (1) and (2) respectively explore the impact of CSR and financialization on corporate innovation before 2014. The coefficients of CSR and financialization are 0.0211 and −1.8040, both significant at the 1% level. In the results of Column (3), there is a positive association between CSR and corporate innovation, while there is a negative association between financialization and corporate innovation. The moderating effect of financialization before 2014 is explored in Column (4), and its results indicate that the financialization of high-polluting firms cannot diminish the contribution of CSR to corporate innovation, which supports H5a. Columns (5) and (6) respectively explore the impact of CSR and financialization on corporate innovation after 2014. The coefficients of CSR and financialization are 0.0134 and −1.4905, respectively significant at the 1% level and 5% level. In the results of Column (7), CSR can promote corporate innovation (0.0132, significant at the 1% level), while financialization can inhibit corporate innovation (−1.3805, significant at the 5% level). Column (8) explores the moderating effect of financialization after 2014, and its results demonstrate that financialization can alleviate the positive association between CSR and corporate innovation, which supports H5b. By comparing the results in different periods, the implementation of environmental policy will not change the impact of CSR or financialization on corporate innovation in high-polluting industries. However, new environmental policies can promote the crowding out effect of financialization on R&D expenditures, thus inhibiting managers’ innovation motivations.

#### 4.2.5. Regression Results of Different CSR Engagements

In resource theory, corporate innovation can only be supported by limited resources in high-polluting industries. As a result, high-polluting firms should not only focus on the environmental benefits of technology upgrades, but also consider the economic benefits of innovation outcomes. Different CSR engagements can present the efforts made by high-polluting firms for stakeholders or shareholders. Considering the benefits from different dimensions, this study discusses the stakeholders-related and shareholders-related activities to explore the impact of different CSR engagements on corporate innovation. For empirical models, this study further uses the CSR score of stakeholders/shareholders to replace the total score of CSR in Equations (1) and (2), and constructs Equations (3) and (4).

(3) $$\begin{array}{ll} Innovation_{i,t}= & \alpha_{0}+\beta_{1}CSR\_ST_{i,t-1}\left(CSR\_SH_{i,t-1}\right)+\beta_{2}FIN_{i,t-1}+\beta_{3}ROA_{i,t-1}\\ & +\beta_{4}Leverage_{i,t-1}+\beta_{5}Age_{i,t-1}+\beta_{6}Asset\;turnover_{i,t-1}\\ & +\beta_{7}Tobin\;Q_{i,t-1}+\beta_{8}Liquidity_{i,t-1}+\beta_{9}Sales_{i,t-1}\\ & +\beta_{10}Independ_{i,t-1}+\beta_{11}Hold_{i,t-1}+Year_{fixed\;effects}\\ & +Industry_{fixed\;effects}+\varepsilon \end{array}$$

(4) $$\begin{array}{ll} Innovation_{i,t}= & \alpha_{0}+\beta_{1}CSR\_ST_{i,t-1}\left(CSR\_SH_{i,t-1}\right)+\beta_{2}FIN_{i,t-1}\\ & +\beta_{3}CSR\_ST_{i,t-1}\left(CSR\_SH_{i,t-1}\right)\times FIN_{i,t-1}+\beta_{4}ROA_{i,t-1}\\ & +\beta_{5}Leverage_{i,t-1}+\beta_{6}Age_{i,t-1}+\beta_{7}Asset\;turnover_{i,t-1}\\ & +\beta_{8}Tobin\;Q_{i,t-1}+\beta_{9}Liquidity_{i,t-1}+\beta_{10}Sales_{i,t-1}\\ & +\beta_{11}Independ_{i,t-1}+\beta_{12}Hold_{i,t-1}+Year_{fixed\;effects}\\ & +Industry_{fixed\;effects}+\varepsilon \end{array}$$

In Equations (3) and (4), $CSR\_ST_{i,t-1}$ represents the CSR score of stakeholders of firm *i* in year *t* − 1. $CSR\_SH_{i,t-1}$ represents the CSR score of shareholders of firm *i* in year *t* − 1.

Just like the baseline results of regression analysis, this study adopts the same empirical method to test the impact of different CSR engagements on corporate innovation. The results of overall samples are reported in Table 8.

In Table 8, Columns (1) and (4) respectively explore the impact of stakeholders-related and shareholders-related activities on corporate innovation. The coefficients of CSR_ST and CSR_SH are 0.0164 and 0.0743, both significant at the 1% level. Columns (2) and (5) respectively explore the impact of different CSR engagements and financialization on corporate innovation. The coefficients of CSR_ST (CSR_SH) and financialization are 0.0163 (0.0741) and −1.5966 (−1.6474), both significant at the 1% level. The results of Columns (1), (2), (4), and (5) can be consistent with those of Table 4, indicating that CSR engagements can promote the technology upgrades of high-polluting firms, and financialization may hinder their innovation activities. Column (3) explores the moderating effect of financialization. The coefficient of interaction (CSR_ST*FIN) is −0.0643, significant at the 5% level, indicating that the financialization of high-polluting firms may relieve the positive association between stakeholders-related activities and corporate innovation. It is surprising that the moderating effect of financialization is not significant in Column (6), so the financialization of high-polluting firms cannot alleviate the positive association between shareholders-related activities and corporate innovation.

In high-polluting industries, SOEs can get more support from local governments, and their managers may not pay more attention to environmental benefits because of the policy advantages. In contrast, Non-SOEs should not only achieve the goal of profit maximization, but also make the contributions to society and environment for their investors. In this situation, the managers of different ownership in high-polluting industries may focus on different CSR engagements because of the demands of stakeholders and the interests of shareholders. Therefore, this study uses the different CSR engagements to test the impact of different ownership on corporate innovation. The empirical results are reported in Table 9.

In Table 9, Columns (1) and (3) respectively explore the impact of CSR_ST (CSR_SH) and financialization on corporate innovation in SOEs. There is a significant positive association between different CSR engagements and corporate innovation, while financialization cannot produce the significant impact on their innovation outcomes. Columns (2) and (4) respectively explore the moderating effect of financialization on different CSR engagements, and their results indicate that financialization can only alleviate the role of stakeholders-related activities in promoting corporate innovation. Furthermore, Columns (5)–(8) respectively explore the impact of CSR_ST (CSR_SH) and financialization on corporate innovation in Non-SOEs. From the results of Columns (5) and (6), there is no significant positive association between the stakeholders-related activities and corporate innovation. Financialization cannot change the impact of stakeholders-related activities on R&D process either. From the results of Columns (7) and (8), there is a significant positive association between the shareholders-related activities and corporate innovation, but the financialization of Non-SOEs cannot have a significant impact on the role of shareholders-related activities in promoting corporate innovation.

Based on the results of Table 6, financial constraints faced by high-polluting firms are important factors in determining the decision of corporate innovation. Therefore, this study further discusses the different CSR engagements of high-polluting firms with different constraints. The empirical results are reported in Table 10.

In Table 10, Columns (1)–(4) respectively explore the impact of CSR engagements and financialization on corporate innovation under the condition of low financial constraints. From the results of Columns (1)–(4), there is a significant positive association between stakeholders-related (shareholders-related) activities and corporate innovation, but the financialization of high-polluting firms cannot significantly influence the R&D activities. It is worth noting that financialization can alleviate the role of different CSR engagements in promoting innovation. Columns (5)–(8) respectively explore the impact of CSR engagements and financialization on corporate innovation under the condition of high financial constraints. Just like the results of Table 6, there is a significant positive association between different CSR engagements and corporate innovation, while the financialization of high-polluting firms has a negative impact on their innovation outcomes.

Different from other policies, environmental policies provide more limitations for high-polluting firms, especially in energy consumption and pollutant emission. Therefore, this study uses different CSR engagements to test the impact of environmental policies on the stakeholders-related and shareholders-related activities. The empirical results are reported in Table 11.

In Table 11, Columns (1)–(4) respectively explore the impact of CSR engagements and financialization on corporate innovation before the promulgation of The Environmental Protection Law of China in 2014. There is a significant positive association between different CSR engagements and corporate innovation, while financialization has a negative impact on the R&D activities of high-polluting firms. Just like the results of Table 7, the moderating effect of financialization is not significant before 2014. Columns (5)–(8) respectively explore the impact of CSR engagements and financialization on corporate innovation during the implementation of environmental policy. It can be seen that the moderating effect of financialization in Column (6) is significant for stakeholders-related activities, while it is not significant for shareholders-related activities in Column (8). This finding suggests that environmental policies may change the relationship between CSR and financialization, especially in stakeholders-related activities, and the managers of high-polluting firms will regard CSR engagements as a special business strategy.

### 4.3. Robustness Tests

According to the findings in Section 4.2, CSR can significantly promote corporate innovation in high-polluting industries, while financialization can inhibit the innovation outcomes of such firms. Patent applications can represent the difference in innovation outcomes among high-polluting firms. In order to ensure the reliability of empirical results, this study uses whether high-polluting firms conduct patent applications to measure corporate innovation, and changes corporate innovation from a continuous variable to a binary variable. In robustness tests, this study conducts the empirical models based on Equations (1) and (2), and uses Logit regression for empirical analysis. The regression results of robustness tests are reported in Table 12.

In Table 2, it can be seen that CSR still promotes corporate innovation in high-polluting industries, while financialization inhibits corporate innovation. These results indicate that the innovation outcomes of high-polluting firms can be influenced by social responsibility activities and capital structure. For the results of Column (4), the financialization of high-polluting firms can alleviate the positive association between CSR and corporate innovation, indicating that the increase of financial assets may gradually reduce managers’ innovation intention.

For different CSR engagements, this study uses the stakeholders-related and shareholders-related scores to replace the total score of CSR, and adopts Logit regression for robustness tests mentioned in Table 12. The robustness results are reported in Table 13.

In Table 13, there is a significant positive association between CSR engagements and corporate innovation, while financialization can still hinder the R&D activities of high-polluting firms. For stakeholders-related activities, the moderating effect of financialization is significant at the 5% level. However, financialization cannot change the role of shareholders-related activities in promoting corporate innovation. Therefore, the findings of robustness tests can support the results in Section 4.2.

## 5. Discussion

High-polluting industries, as one of the main sources of environmental pollutant, play an important role in economic growth, particularly in the developing countries. In order to mitigate environmental damages caused by the operations of high-polluting firms, corporate innovation is an important approach to improve the efficiency of resource utilization and reduce the emission of air pollutant. The R&D activities depend on a large number of internal resources, and high-polluting firms need to resolve the shortage of funds. On the one hand, a variety of corporate practices in environmental protection can show the efforts made by high-polluting firms to reduce the accidents of environmental pollution and damage. On the other hand, high-polluting firms can use some kinds of financial assets to relieve the shortage of funds, but these assets may have a crowd out effect on R&D expenditures. Because of high profits, some managers of high-polluting firms tend to invest more in financial assets to meet the demands of shareholders, thus gradually ignoring the practical value of innovation outcomes. The empirical results of this study are consistent with the findings of Hasan et al. and Tori and Onaran [14,39].

This study discusses corporate innovation from different dimensions, including ownership, financial constraint, and environmental policy. In terms of ownership, SOEs in high-polluting industries can use sufficient resources to support their R&D activities, but their financial assets may weaken managers’ motivations for innovation. Although Non-SOEs need to use external financing and financial assets to alleviate the shortage of funds, their innovation intention cannot be affected by financialization directly. This suggests that CSR dominates R&D activities in non-state-owned enterprises, which is similar to the findings of Ji and Miao [53]. As for financial constraints, high-polluting firms with low financial constraints can use sufficient resources to promote the activities of technological innovation. However, increasing the proportion of financial assets may lead to the decrease of innovation motivations, which supports the findings of Zhang and Zheng [54]. Although high-polluting firms with high financial constraints need to use financial assets to improve their profitability, CSR still plays a dominant role in corporate innovation. With regard to environmental policy, the aim of environmental policy is to balance the association between high-polluting firms and ecological environment, and some policies may make high-polluting firms choose two strategies: reducing production or updating technology. It is argued that technological innovation will be based on great time costs, but reducing production can be implemented in a short time without considering market demands [47]. For this reason, some high-polluting firms often use financial assets to meet the needs of shareholders in the process of reducing production, which can also reduce managers’ innovation intention.

Because of limited resources, managers cannot match the demands of stakeholders and the interests of shareholders at the same time. This study also discusses the impact of different CSR engagements on corporate innovation. Both the stakeholders-related and shareholders-related activities will promote corporate innovation, indicating that China’s high-polluting firms do not ignore the core idea of CSR, namely responsibility and sustainability. Compared with stakeholders-related activities, the role of shareholders-related activities is not sensitive to financialization in promoting corporate innovation. This finding is consistent with the results of Li and Wu [13], suggesting that some CSR activities are always being regarded as a kind of business strategy in high-polluting industries. In addition, the shareholders-related activities of SOEs cannot be influenced by investments in financial assets, while their stakeholders-related activities are sensitive to financialization. This performance gap demonstrates that the policy and asset advantages are more likely to transfer SOEs’ CSR engagements to business strategies, and this may increase their economic benefits with decreasing environmental benefits from innovation outcomes. Furthermore, the implementation of environmental policies can also change managers’ attention to CSR engagements in high-polluting industries, which is similar to the results of Allen and Craig [2]. Based on the discussions of different CSR engagements, there are some obvious performance gaps between stakeholders-related and shareholders-related activities, and these differences demonstrate that CSR engagements may not always meet the demands of stakeholders in high-polluting industries.

## 6. Conclusions and Recommendations

### 6.1. Conclusions

Corporate innovation in high-polluting industries is an important approach to protect ecological environment, and is also the main driving force for the sustainable development of regional economy. In resource theory, high-polluting firms need to use limited resources to support technological innovation for improving the energy consumption and reducing pollutant emissions. Compared with other manufacturing firms, high-polluting firms need to use innovation outcomes to deliver information about social responsibility practices to stakeholders, and also rely on some investments in financial assets to meet the demands of shareholders. On the one hand, CSR can help high-polluting firms to get more recognition from investors, thereby expanding financing channels and reducing financing costs. On the other hand, high-polluting firms can gain high profits in the short term by investing in financial and real estate industries. It is worth noting that managers’ attention to CSR engagements can be changed by financialization, and some CSR engagements may be regarded as a business strategy. Therefore, exploring the impact of CSR and financialization on corporate innovation will become a determining factor in the sustainable ability of high-polluting firms.

This study discusses the association among CSR, financialization, and corporate innovation based on China’s listed companies in high-polluting industries. CSR can promote the technological innovation of high-polluting firms, and this reveals their contributions to environmental protection and social stability. On the contrary, financialization can reduce the R&D expenditures of high-polluting firms, which may hinder corporate innovation. The moderating effect of financialization demonstrates that more and more investments in financial assets can change the dominant position of CSR in promoting corporate innovation, and then may weaken the managers’ motivation of technology upgrades. Furthermore, the stakeholders-related activities of high-polluting firms are more sensitive to financialization than shareholders-related activities. This performance gap indicates that some CSR engagements may be the business strategy of high-polluting firms, not just contributions to environment and society.

In the Chinese market, SOEs with capital and policy advantages, play an important role in economic growth. SOEs in high-polluting industries need to maintain the image of local governments through some social responsibility activities. Although the sufficient funds of SOEs can relieve the crowding out effect of financialization on R&D expenditures, high profits of investments in financial assets may also alleviate managers’ innovation intention. It is interesting that some SOEs’ practices for shareholders are not sensitive to financialization, and this kind of CSR engagements can promote the innovation outcomes of high-polluting firms all the time. In contrast, Non-SOEs need to rely on financial assets to improve their profitability, and financialization has a crowding out effect on their R&D expenditures. The recognition of investors is important to Non-SOEs, so that their innovation decisions may be mainly driven by environmental benefits. Moreover, financialization will have little crowding out effect on R&D expenditures under the condition of low financial constraints, but these financial assets can change the role of CSR engagements in technological innovation. Under the condition of high financial constraints, financialization will reduce some investments in R&D activities, but CSR activities can still play a dominant role in technology upgrading. In addition, the implementation of environmental policies may change managers’ attention to CSR engagements. Faced with environmental policies, financialization cannot change the role of shareholders-related activities in promoting innovation, and high-polluting firms prefer obtaining more environmental benefits from innovation outcomes.

The innovation outcomes of high-polluting firms can directly change the impact of their operations on ecological environment. Based on the signal theory and resource theory, this study demonstrates that CSR can play an important role in promoting corporate innovation, but CSR engagements will reveal the different efforts made by high-polluting firms. This performance gap may be driven by managers’ attention to CSR activities. In addition, high-polluting firms need to relieve the crowding out effect of financial assets on R&D expenditures, and pay more attention to the importance of technological innovation in their long-term development.

### 6.2. Recommendations and Limitations

According to the theoretical analysis and empirical analysis, CSR can promote the innovation outcomes of high-polluting firms, while financialization can hinder corporate innovation. These findings can help high-polluting industries to achieve a technological transformation, and enhance the sustainable ability of high-polluting firms. This study may have the following implications:

First, high-polluting firms need to maintain the dominant position of CSR in innovation. CSR has long been regarded as an important factor in promoting corporate innovation, and can improve the association between high-polluting firms and ecological environment. However, some high-polluting firms may choose to invest more and more in financial assets for obtaining high profits. In this situation, the managers of high-polluting firms may ignore the importance of CSR. Compared with other industries, the CSR engagements of high-polluting firms can directly affect their productions and operations. Therefore, the dominant position of CSR in innovation can be a key factor for high-polluting firms to achieve the goal of sustainable development.

Second, high-polluting firms need to alleviate the impact of financialization on profitability. Financial assets can bring high profits in a short time, but this kind of asset is accompanied by high risks. Therefore, the high proportion of financial assets can directly increase the financial risk and operational risk of high-polluting firms, and may even form the systemic risk in highly polluting industries. Accordingly, high-polluting firms need to pay attention to the proportion of financial assets, and make great efforts to corporate innovation.

This study has several limitations, and needs to be improved in the future research. Climate change could be an interesting and meaningful context, under which the issues of corporate innovation in high-polluting industries could be better examined. Climate change could be a time-series moderate factor that could have impact on the associations among corporate innovations, CSR, and financialization, or it could be a result from the joint effects of corporate innovations, CSR, and financialization. Moreover, in the case of the effects on climate, the impact may only be visible after many decades. It is hard to capture climate changes in our short window studies. So we call for further empirical evidence. A similar limitation is on the concerns of accountability, particularly as related to long-term pollution, which may not be detectable immediately on the ground.

In terms of research sample, this study chooses A-share listed companies in the Chinese market, and other listed companies on Small and Medium board Enterprise Board and Growth Enterprises Market are removed. The research results are focused on high-polluting firms with large assets, and the findings might not completely represent the characteristics of high-polluting industries. In terms of research design, this study only considers the linear effect of CSR and financialization on corporate innovation. However, there may be a non-linear relationship between CSR or financialization and corporate innovation, and this limitation may influence the practicability of empirical findings. Even so, our findings should be thought-provoking evidence on the corporate innovations, especially in the high-pollution industries.

## Figures and Tables

**Table 1:** Variable definitions.

Variables	Type	Definition
Innovation	Dependent Variable	Corporate innovation is calculated by the natural logarithm of patent applications plus one.
CSR	Independent Variables	CSR is measured as CSR overall score from Hexun CSR Database.
FIN		FIN is calculated as the financial assets divided by the total assets.
ROA	Control Variables	ROA is calculated as the net income divided by the total assets.
Leverage		Leverage is calculated as the total debts divided by the total assets.
Age		Age is measured by the duration from the year the firm was established to the sample year.
Asset turnover		Asset turnover is calculated as the total revenues divided by the total assets.
Tobin Q		Tobin Q is calculated as the market value divided by the total assets.
Liquidity		Liquidity is calculated as the current assets divided by the total assets.
Sales		Sales is calculated as the growth rate of operation revenues.
Independ		Independ is calculated as the number of independent directors divided by the total number of directors on board.
Hold		Hold is calculated as the sharing ratio of the top three shareholders.

**Table 2:** Descriptive statistics.

Variable	Observations	Mean	Std. Dev.	Min	Median	Max
Innovation	3428	2.1725	1.7566	0.0000	2.1972	6.6796
CSR	3428	28.8457	21.0923	−2.8300	22.3500	78.2600
FIN	3428	0.0357	0.0699	0.0000	0.0006	0.4088
ROA	3428	0.0387	0.0666	−0.1768	0.0281	0.2650
Leverage	3428	0.5066	0.2110	0.0559	0.5133	0.9934
Age	3428	18.7876	4.2329	10.0000	19.0000	30.0000
Asset turnover	3428	0.7028	0.4487	0.0946	0.6096	2.4732
Tobin Q	3428	2.1026	1.4900	0.8935	1.5857	9.3343
Liquidity	3428	0.4361	0.1973	0.0610	0.4311	0.8787
Sales	3428	0.1666	0.4696	−0.4893	0.0901	3.3236
Independ	3428	0.3702	0.0516	0.3000	0.3333	0.5714
Hold	3428	0.1919	0.1455	0.0082	0.1617	0.7056

**Table 3:** Pearson correlation matrix.

	**Innovation**	**CSR**	**FIN**	**ROA**	**Leverage**	**Age**	**VIF**
Innovation	1						
CSR	0.175 ***	1					1.25
FIN	−0.124 ***	−0.041 **	1				1.07
ROA	0.045 ***	0.374 ***	0.046 ***	1			1.62
Leverage	0.028	−0.172 ***	−0.134 ***	−0.459 ***	1		1.40
Age	−0.002	−0.184 ***	0.136 ***	−0.065 ***	−0.001	1	1.14
Asset turnover	0.147 ***	0.056 ***	−0.134 ***	0.145 ***	−0.039 **	−0.101 ***	1.16
Tobin Q	−0.187 ***	−0.068 ***	0.104 ***	0.256 ***	−0.271 ***	0.068 ***	1.29
Liquidity	−0.033 *	0.005	0.028 *	0.248 ***	−0.309 ***	−0.044 **	1.37
Sales	−0.035 **	0.056 ***	−0.002	0.213 ***	0.006	−0.029 *	1.07
Independ	−0.005	0.009	0.014	0.012	−0.046 ***	−0.052 ***	1.02
Hold	0.095 ***	0.170 ***	−0.130 ***	0.091 ***	0.018	−0.288 ***	1.19
	**Asset Turnover**	**Tobin Q**	**Liquidity**	**Sales**	**Independ**	**Hold**	
Asset turnover	1						
Tobin Q	0.020	1					
Liquidity	0.285 ***	0.373 ***	1				
Sales	0.101 ***	0.010	0.047 ***	1			
Independ	−0.015	0.042 **	0.011	0.008	1		
Hol	0.133 ***	−0.159 ***	−0.090 ***	0.083 ***	0.119 ***	1	

Note: ***, **, * represent the significance at the level of 1%, 5%, 10% respectively.

**Table 4:** Baseline results of overall samples.

Variable	Innovation					
	(1)	(2)	(3)	(4)	(5)	(6)
CSR	0.0187 ***		0.0165 ***		0.0164 ***	0.0160 ***
	(12.85)		(10.53)		(10.52)	(10.28)
FIN		−2.2325 ***		−1.6819 ***	−1.5928 ***	−1.6726 ***
		(−5.10)		(−3.9)	(−3.73)	(−3.84)
CSR*FIN						−0.0497 **
						(−2.28)
Leverage			0.7790 ***	0.5765 ***	0.7163 ***	0.7718 ***
			(4.53)	(3.3)	(4.14)	(4.12)
Age			−0.0118	−0.0128	−0.0104	−0.0114
			(−1.34)	(−1.44)	(−1.19)	(−1.31)
Asset turnover			0.3878 ***	0.3530 ***	0.3621 ***	0.3571 ***
			(5.21)	(4.63)	(4.90)	(4.82)
Tobin Q			−0.1847 ***	−0.2162 ***	−0.1812 ***	−0.1837 ***
			(−8.15)	(−9.67)	(−7.96)	(−8.05)
Liquidity			−0.8688 ***	−1.0307 ***	−0.8775 ***	−0.8597 ***
			(−4.52)	(−5.29)	(−4.58)	(−4.49)
Sales			−0.0882	−0.1157 *	−0.0858	−0.0835
			(−1.39)	(−1.78)	(−1.36)	(−1.32)
ROA			2.2296 ***	4.3387 ***	2.2572 ***	2.2487 ***
			(3.98)	(7.88)	(4.05)	(4.03)
Independ			−0.5263	−0.4799	−0.5101	−0.4517
			(−0.81)	(−0.74)	(−0.78)	(−0.69)
Hold			−0.5075 *	−0.3881	−0.5779 **	−0.5948 **
			(−1.87)	(−1.4)	(−2.13)	(−2.19)
Constant	1.4199 ***	2.1090 ***	1.9265 ***	2.6437 ***	1.9921 ***	2.0001 ***
	(8.17)	(11.44)	(5.60)	(7.57)	(5.80)	(5.83)
YearIndustry	yes	yes	yes	yes	yes	yes
	yes	yes	yes	yes	yes	yes
Observations	2915	2915	2915	2915	2915	2915
Adjusted R^2^	0.2009	0.1591	0.2431	0.2157	0.2464	0.2478

Note: t statistics are in parentheses; ***, **, * represent the significance at the level of 1%, 5%, 10% respectively.

**Table 5:** Regression results of state-owned enterprises (SOEs) and non-SOEs.

Variable	Innovation							
	SOEs				Non-SOEs			
	(1)	(2)	(3)	(4)	(5)	(6)	(7)	(8)
CSR	0.0145 ***		0.0145 ***	0.0136 ***	0.0074 **		0.0070 **	0.0070 **
	(8.45)		(8.44)	(7.76)	(2.36)		(2.22)	(2.22)
FIN		0.5583	0.4762	0.7140		−2.3975 ***	−2.3459 ***	−2.3536 ***
		(0.90)	(0.74)	(1.12)		(−5.14)	(−5.02)	(−4.56)
CSR*FIN				−0.0731 **				−0.0014
				(−2.49)				(−0.05)
Leverage	1.0820 ***	0.9066 ***	1.1116 ***	1.1075 ***	0.1609	0.1917	0.1917	0.1921
	(5.15)	(4.24)	(5.24)	(5.21)	(0.55)	(0.65)	(0.66)	(0.66)
Age	0.0174	0.0165	0.0177 *	0.0168	−0.0079	−0.0025	−0.0039	−0.0039
	(1.63)	(1.51)	(1.65)	(1.51)	(−0.56)	(−0.17)	(−0.28)	(−0.28)
Asset turnover	−0.0051	−0.0275	−0.0018	−0.0044	0.4934 ***	0.3985 ***	0.4032 ***	0.4031 ***
	(−0.06)	(−0.32)	(−0.02)	(−0.05)	(3.27)	(2.62)	(2.68)	(2.68)
Tobin Q	−0.2658 ***	−0.2910 ***	−0.2675 ***	−0.2668 ***	−0.1210 ***	−0.1397 ***	−0.1238 ***	−0.1239 ***
	(−8.01)	(−8.76)	(−8.10)	(−7.95)	(−4.13)	(−4.87)	(−4.24)	(−4.24)
Liquidity	−0.4605 **	−0.5845 **	−0.4475 *	−0.4059 *	−0.7614 **	−0.8282 ***	−0.7759 **	−0.7761 **
	(−1.97)	(−2.42)	(−1.91)	(−1.73)	(−2.41)	(−2.67)	(−2.49)	(−2.49)
Sales	−0.0507	−0.0555	−0.0517	−0.0485	0.0248	0.0050	0.0206	0.0207
	(−0.59)	(−0.62)	(−0.60)	(−0.56)	(0.31)	(0.06)	(0.26)	(0.26)
ROA	4.7901 ***	6.7698 ***	4.8003 ***	4.7634 ***	2.4742 ***	3.5002 ***	2.6486 ***	2.6486 ***
	(6.76)	(9.73)	(6.78)	(6.70)	(3.12)	(4.74)	(3.30)	(3.30)
Independ	−0.4485	−0.4823	−0.4670	−0.4660	0.7386	0.6815	0.5547	0.5567
	(−0.64)	(−0.68)	(−0.67)	(−0.64)	(0.68)	(0.65)	(0.52)	(0.52)
Hold	1.1786 ***	1.4170 ***	1.1916 ***	1.1711 ***	−4.0186 ***	−4.0740 ***	−4.1056 ***	−4.1060 ***
	(3.69)	(4.31)	(3.74)	(3.68)	(−10.57)	(−10.90)	(−10.90)	(−10.89)
Constant	0.9197 **	1.4923 ***	0.8964 **	0.9193 **	1.8705 ***	2.5105 ***	2.2896 ***	2.2895 ***
	(2.30)	(3.63)	(2.23)	(2.29)	(2.71)	(3.69)	(3.31)	(3.31)
Year	yes	yes	yes	yes	yes	yes	yes	yes
Industry	yes	yes	yes	yes	yes	yes	yes	yes
Observations	1974	1974	1974	1974	941	941	941	941
Adjusted R^2^	0.3348	0.3097	0.3350	0.3374	0.3114	0.3180	0.3221	0.3221

Note: t statistics are in parentheses; ***, **, * represent the significance at the level of 1%, 5%, 10% respectively.

**Table 6:** Regression results of different constraints.

Variable	Innovation							
	Low Constraints				High Constraints			
	(1)	(2)	(3)	(4)	(5)	(6)	(7)	(8)
CSR	0.0179 ***		0.0178 ***	0.0167 ***	0.0098 ***		0.0096 ***	0.0096 ***
	(8.09)		(8.09)	(7.41)	(4.46)		(4.42)	(4.44)
FIN		−0.6407	−0.6177	−0.4726		−2.2725 ***	−2.2225 ***	−2.1601 ***
		(−0.88)	(−0.85)	(−0.66)		(−4.35)	(−4.30)	(−4.05)
CSR*FIN				−0.0931 **				0.0154
				(−2.59)				(0.49)
Leverage	0.5848 **	0.4162	0.5703 **	0.5343 **	0.5560 **	0.3272	0.4093 *	0.4027*
	(2.24)	(1.55)	(2.19)	(2.06)	(2.37)	(1.37)	(1.71)	(1.67)
Age	0.0474 **	0.0630 ***	0.0480 **	0.0467 **	0.0183	0.0193	0.0202	0.0206
	(2.10)	(2.72)	(2.12)	(2.06)	(1.17)	(1.21)	(1.30)	(1.32)
Asset turnover	0.2354 **	0.1804	0.2304 **	0.2343 **	0.4618 ***	0.4561 ***	0.4341 ***	0.4372 ***
	(2.09)	(1.52)	(2.04)	(2.07)	(4.60)	(4.59)	(4.40)	(4.42)
Tobin Q	−0.2521 ***	−0.3050 ***	−0.2508 ***	−0.2529 ***	−0.1311 ***	−0.1436 ***	−0.1287 ***	−0.1278 ***
	(−7.06)	(−8.83)	(−7.01)	(−6.98)	(−4.26)	(−4.68)	(−4.15)	(−4.12)
Liquidity	−1.1188 ***	−1.2870 ***	−1.1265 ***	−1.0905 ***	−0.3402	−0.4188 *	−0.3595	−0.3641
	(−3.71)	(−4.15)	(−3.73)	(−3.61)	(−1.41)	(−1.76)	(−1.52)	(−1.54)
Sales	−0.0559	−0.0863	−0.0556	−0.0541	−0.0354	−0.0528	−0.0402	−0.0414
	(−0.58)	(−0.88)	(−0.58)	(−0.57)	(−0.50)	(−0.73)	(−0.57)	(−0.59)
ROA	2.3770 ***	4.8355 ***	2.4160 ***	2.2878 ***	2.6029 ***	3.6061 ***	2.5064 ***	2.4902 ***
	(2.92)	(6.11)	(2.98)	(2.81)	(3.48)	(4.98)	(3.38)	(3.35)
Independ	−2.2628 ***	−2.4791 ***	−2.2438 ***	−2.2203 **	2.0668 **	2.0306 **	1.9301 **	1.9293 **
	(−2.62)	(−2.80)	(−2.60)	(−2.56)	(2.17)	(2.17)	(2.04)	(2.03)
Hold	−0.6979*	−0.5019	−0.7206*	−0.7170 *	0.0898	0.0244	−0.0218	−0.0073
	(−1.80)	(−1.26)	(−1.85)	(−1.84)	(0.26)	(0.07)	(−0.06)	(−0.02)
Constant	2.4435 ***	3.1501 ***	2.4505 ***	2.4788 ***	−1.4794 ***	−1.0750 ***	−1.2657 ***	−1.2815 ***
	(4.59)	(5.76)	(4.60)	(4.65)	(−3.13)	(−2.24)	(−2.67)	(−2.70)
Year	yes	yes	yes	yes	yes	yes	yes	yes
Industry	yes	yes	yes	yes	yes	yes	yes	yes
Observations	1471	1471	1471	1471	1444	1444	1444	1444
Adjusted R^2^	0.3177	0.2857	0.3180	0.3215	0.2588	0.2562	0.2676	0.2677

Note: t statistics are in parentheses; ***, **, * represent the significance at the level of 1%, 5%, 10% respectively.

**Table 7:** Regression results of different periods.

Variable	Innovation							
	Before 2014				After 2014			
	(1)	(2)	(3)	(4)	(5)	(6)	(7)	(8)
CSR	0.0211 ***		0.0211 ***	0.0206 ***	0.0134 ***		0.0132 ***	0.0128 ***
	(10.00)		(10.02)	(9.80)	(5.84)		(5.79)	(5.67)
FIN		−1.8040 ***	−1.7557 ***	−1.6060**		−1.4905 **	−1.3805 **	−1.6608 ***
		(−2.82)	(−2.73)	(−2.58)		(−2.59)	(−2.42)	(−2.82)
CSR*FIN				−0.0481				−0.0649 **
				(−1.46)				(−2.28)
Leverage	0.5695 **	0.3295	0.5074 **	0.5038 **	0.8189 ***	0.6681 ***	0.7635 ***	0.7526 ***
	(2.30)	(1.24)	(2.04)	(2.03)	(3.42)	(2.80)	(3.17)	(3.12)
Age	−0.0188	−0.0225*	−0.0165	−0.0172	−0.0034	−0.0038	−0.0028	−0.0047
	(−1.51)	(−1.73)	(−1.35)	(−1.40)	(−0.28)	(−0.31)	(−0.23)	(−0.39)
Asset turnover	0.4542 ***	0.3671 ***	0.4180 ***	0.4194 ***	0.3785 ***	0.3683 ***	0.3609 ***	0.3523 ***
	(4.24)	(3.20)	(3.91)	(3.92)	(3.67)	(3.55)	(3.51)	(3.41)
Tobin Q	−0.1188 ***	−0.1836 ***	−0.1196 ***	−0.1168 ***	−0.2277 ***	−0.2441 ***	−0.2218 ***	−0.2282 ***
	(−3.19)	(−4.69)	(−3.21)	(−3.12)	(−8.00)	(−8.74)	(−7.72)	(−8.00)
Liquidity	−0.9999 ***	−1.2742 ***	−0.9677 ***	−0.9755 ***	−0.7784 ***	−0.8706 ***	−0.8084 ***	−0.7720 ***
	(−3.71)	(−4.59)	(−3.61)	(−3.64)	(−2.86)	(−3.15)	(−2.97)	(−2.84)
Sales	−0.1321	−0.1583 *	−0.1227	−0.1218	−0.0541	−0.0885	−0.0542	−0.0496
	(−1.47)	(−1.67)	(−1.38)	(−1.36)	(−0.64)	(−1.02)	(−0.64)	(−0.59)
ROA	2.1955 **	4.9838 ***	2.2183 ***	2.1904 **	1.8763 **	3.5741 ***	1.9189 **	1.8954 **
	(2.58)	(5.67)	(2.61)	(2.57)	(2.45)	(4.87)	(2.53)	(2.49)
Independ	−0.3900	−0.5128	−0.3391	−0.2437	−0.6818	−0.5559	−0.6778	−0.6471
	(−0.44)	(−0.57)	(−0.38)	(−0.28)	(−0.73)	(−0.60)	(−0.73)	(−0.69)
Hold	−0.8909 **	−0.5864	−0.9526 ***	−0.9598 ***	−0.2364	−0.2188	−0.3075	−0.3414
	(−2.47)	(−1.54)	(−2.64)	(−2.66)	(−0.60)	(−0.55)	(−0.77)	(−0.86)
Constant	1.8135 ***	2.9513 ***	1.8475 ***	1.8168 ***	1.9069 ***	2.4334	1.9735 ***	2.0142 ***
	(3.50)	(5.52)	(3.57)	(3.51)	(4.08)	(5.18)	(4.23)	(4.33)
Year	yes	yes	yes	yes	yes	yes	yes	yes
Industry	yes	yes	yes	yes	yes	yes	yes	yes
Observations	1261	1261	1261	1261	1654	1654	1654	1654
Adjusted R^2^	0.2633	0.2070	0.2671	0.2684	0.2347	0.2201	0.2372	0.2393

Note: t statistics are in parentheses; ***, **, * represent the significance at the level of 1%, 5%, 10% respectively.

**Table 8:** Different CSR engagements of overall samples.

Variable	Innovation					
	(1)	(2)	(3)	(4)	(5)	(6)
CSR_ST	0.0164 ***	0.0163 ***	0.0158 ***			
	(9.44)	(9.41)	(9.15)			
CSR_SH				0.0743 ***	0.0741 ***	0.0738 ***
				(9.85)	(9.89)	(9.86)
FIN		−1.5966 ***	−1.7051 ***		−1.6474 ***	−1.6477 ***
		(−3.72)	(−3.90)		(−3.92)	(−3.91)
CSR_ST*FIN			−0.0643 **			
			(−2.57)			
CSR_SH*FIN						−0.0724
						(−1.02)
Leverage	0.7211 ***	0.6585 ***	0.6479 ***	0.9019 ***	0.8371 ***	0.8425 ***
	(4.17)	(3.79)	(3.73)	(5.20)	(4.80)	(4.84)
Age	−0.0126	−0.0112	−0.0124	−0.0110	−0.0095	−0.0096
	(−1.42)	(−1.28)	(−1.42)	(−1.23)	(−1.08)	(−1.10)
Asset turnover	0.3922 ***	0.3664 ***	0.3636 ***	0.3586 ***	0.3322 ***	0.3278 ***
	(5.25)	(4.94)	(4.89)	(4.76)	(4.44)	(4.37)
Tobin Q	−0.1976 ***	−0.1940 ***	−0.1954 ***	−0.1594 ***	−0.1556 ***	−0.1577 ***
	(−8.70)	(−8.51)	(−8.56)	(−7.12)	(−6.91)	(−6.95)
Liquidity	−0.8773 ***	−0.8860 ***	−0.8669 ***	−0.9904 ***	−0.9984 ***	−0.9932 ***
	(−4.54)	(−4.61)	(−4.52)	(−5.13)	(−5.19)	(−5.17)
Sales	−0.0932	−0.0908	−0.0866	−0.0947	−0.0921	−0.0932
	(−1.47)	(−1.44)	(−1.37)	(−1.46)	(−1.43)	(−1.45)
ROA	3.4944 ***	3.5153 ***	3.4890 ***	−1.4693 *	−1.4370 *	−1.4424 *
	(6.44)	(6.52)	(6.45)	(−1.95)	(−1.91)	(−1.92)
Independ	−0.5550	−0.5385	−0.4744	−0.3759	−0.3598	−0.3446
	(−0.85)	(−0.83)	(−0.73)	(−0.58)	(−0.55)	(−0.53)
Hold	−0.4526 *	−0.5233 *	−0.5473 **	−0.5657 **	−0.6389 **	−0.6382 **
	(−1.66)	(−1.92)	(−2.01)	(−2.08)	(−2.34)	(−2.34)
Constant	2.1450 ***	2.2100 ***	2.2166 ***	1.6050 ***	1.6717 ***	1.6723 ***
	(6.25)	(6.45)	(6.47)	(4.50)	(4.70)	(4.70)
YearIndustry	yes	yes	yes	yes	yes	yes
	yes	yes	yes	yes	yes	yes
Observations	2915	2915	2915	2915	2915	2915
Adjusted R^2^	0.2375	0.2408	0.2424	0.2378	0.2414	0.2416

Note: t statistics are in parentheses; ***, **, * represent the significance at the level of 1%, 5%, 10% respectively.

**Table 9:** Different CSR activities of SOEs and Non-SOEs.

Variable	Innovation							
	SOEs				Non-SOEs			
	(1)	(2)	(3)	(4)	(5)	(6)	(7)	(8)
CSR_ST	0.0146 ***	0.0133 ***			0.0035	0.0035		
	(7.73)	(6.97)			(0.99)	(0.99)		
CSR_SH			0.0672 ***	0.0680 ***			0.0659 ***	0.0667 ***
			(7.26)	(7.27)			(5.58)	(5.56)
FIN	0.4998	0.8173	0.4503	0.4309	−2.3816 ***	−2.3636 ***	−2.1886 ***	−2.1857 ***
	(0.78)	(1.29)	(0.71)	(0.69)	(−5.09)	(−4.34)	(−4.63)	(−4.61)
CSR_ST*FIN		−0.1077 ***				0.0032		
		(−3.38)				(0.08)		
CSR_SH*FIN				0.0660				−0.0614
				(0.65)				(−0.83)
Leverage	1.0601 ***	1.0503 ***	1.1519 ***	1.1492 ***	0.1834	0.1832	0.3480	0.3596
	(4.96)	(4.91)	(5.53)	(5.51)	(0.62)	(0.62)	(1.23)	(1.28)
Age	0.0164	0.0146	0.0218 **	0.0220 **	−0.0030	−0.0030	−0.0056	−0.0056
	(1.53)	(1.36)	(2.04)	(2.06)	(−0.21)	(−0.21)	(−0.40)	(−0.40)
Asset turnover	0.0033	0.0058	−0.0500	−0.0461	0.4003 ***	0.4005 ***	0.4091 ***	0.4073 ***
	(0.04)	(0.07)	(−0.60)	(−0.56)	(2.64)	(2.64)	(2.80)	(2.78)
Tobin Q	−0.2757 ***	−0.2744 ***	−0.2490 ***	−0.2490 ***	−0.1349 ***	−0.1348 ***	−0.0776 ***	−0.0800 ***
	(−8.26)	(−8.12)	(−7.74)	(−7.79)	(−4.67)	(−4.66)	(−2.64)	(−2.72)
Liquidity	−0.4565 *	−0.4010 *	−0.5418 **	−0.5457 **	−0.8021 **	−0.8020 **	−0.8255 ***	−0.8282 ***
	(−1.94)	(−1.71)	(−2.29)	(−2.31)	(−2.57)	(−2.57)	(−2.72)	(−2.72)
Sales	−0.0482	−0.0420	−0.0687	−0.0678	0.0105	0.0103	0.0492	0.0489
	(−0.56)	(−0.49)	(−0.77)	(−0.76)	(0.13)	(0.13)	(0.61)	(0.60)
ROA	6.0041 ***	5.9258 ***	1.0598	1.0551	3.3092 ***	3.3095 ***	−1.0134	−1.0365
	(8.81)	(8.66)	(1.02)	(1.02)	(4.38)	(4.38)	(−1.10)	(−1.12)
Independ	−0.4815	−0.4427	−0.4147	−0.4101	0.6164	0.6135	0.6875	0.7249
	(−0.68)	(−0.63)	(−0.60)	(−0.59)	(0.50)	(0.57)	(0.64)	(0.67)
Hold	1.2469 ***	1.2193 ***	1.1461 ***	1.1485 ***	−4.0814 ***	−4.0801 ***	−4.2393 ***	−4.2382 ***
	(3.90)	(3.82)	(3.57)	(3.58)	(−10.87)	(−10.84)	(−11.31)	(−11.30)
Constant	1.0726 ***	1.0833 ***	0.6625	0.6501	2.4469 ***	2.4463 ***	1.6348 **	1.6190 **
	(2.66)	(2.69)	(1.62)	(1.59)	(3.58)	(3.57)	(2.27)	(2.24)
Year	yes	yes	yes	yes	yes	yes	yes	yes
Industry	yes	yes	yes	yes	yes	yes	yes	yes
Observations	1974	1974	1974	1974	941	941	941	941
Adjusted R^2^	0.3313	0.3352	0.3281	0.3282	0.3188	0.3188	0.3418	0.3421

Note: t statistics are in parentheses; ***, **, * represent the significance at the level of 1%, 5%, 10% respectively.

**Table 10:** Different CSR engagements with different constraints.

Variable	Innovation							
	Low Constraints				High Constraints			
	(1)	(2)	(3)	(4)	(5)	(6)	(7)	(8)
CSR_ST	0.0178 ***	0.0166 ***			0.0094 ***	0.0094 ***		
	(7.37)	(6.75)			(3.92)	(3.93)		
CSR_SH			0.0749 ***	0.0726 ***			0.0467 ***	0.0466 ***
			(6.76)	(6.51)			(4.45)	(4.44)
FIN	−0.5222	−0.5706	−1.0042	−0.6050	−2.2646 ***	−2.2416 ***	−2.0694 ***	−1.9810 ***
	(−0.72	(−0.79)	(−1.39)	(−0.85)	(−4.37)	(−4.18)	(−3.99)	(−3.83)
CSR_ST*FIN		−0.1013 **				0.0081		
		(−2.54)				(0.22)		
CSR_SH*FIN				−0.2129 *				0.0740
				(−1.68)				(0.79)
Leverage	0.5046 *	0.4639 *	0.6999 ***	0.7025 ***	0.3677	0.3651	0.5207 **	0.5120 **
	(1.92)	(1.77)	(2.65)	(2.68)	(1.53)	(1.52)	(2.14)	(2.09)
Age	0.0503 **	0.0487 **	0.0523 **	0.0524 **	0.0208	0.0210	0.0163	0.0160
	(2.22)	(2.14)	(2.28)	(2.28)	(1.34)	(1.35)	(1.03)	(1.02)
Asset turnover	0.2358 **	0.2429 **	0.1581	0.1514	0.4374 ***	0.4382 ***	0.4404 ***	0.4481 ***
	(2.07)	(2.12)	(1.38)	(1.32)	(4.43)	(4.43)	(4.43)	(4.50)
Tobin Q	−0.2678 ***	−0.2686 ***	−0.2288 ***	−0.2325 ***	−0.1352 ***	−0.1351 ***	−0.1112 ***	−0.1088 ***
	(−7.54)	(−7.51)	(−6.30)	(−6.28)	(−4.35)	(−4.35)	(−3.63)	(−3.54)
Liquidity	−1.1327 ***	−1.0869 ***	−1.2636 ***	−1.2720 ***	−0.3616	−0.3625	−0.4166 *	−0.4310 *
	(−3.73)	(−3.58)	(−4.14)	(−4.16)	(−1.53)	(−1.53)	(−1.77)	(−1.83)
Sales	−0.0618	−0.0565	−0.0592	−0.0658	−0.0420	−0.0426	−0.0438	−0.0441
	(−0.65)	(−0.60)	(−0.58)	(−0.65)	(−0.59)	(−0.60)	(−0.63)	(−0.63)
ROA	3.8420 ***	3.7340 ***	−1.2584	−1.3529	3.2070 ***	3.2041 ***	0.1584	0.1360
	(4.90)	(4.74)	(−1.12)	(−1.20)	(4.49)	(4.49)	(0.15)	(0.13)
Independ	−2.2743 ***	−2.2371 **	−2.3517 ***	−2.3602 ***	1.9118 **	1.9135 **	2.1149 **	2.0943 **
	(−2.63)	(−2.58)	(−2.69)	(−2.69)	(2.02)	(2.02)	(2.25)	(2.23)
Hold	−0.6719 *	−0.6759 *	−0.7169 *	−0.7052 *	0.0101	0.0172	−0.1286	−0.1228
	(−1.73)	(−1.73)	(−1.83)	(−1.81)	(0.03)	(0.05)	(−0.38)	(−0.36)
Constant	2.6529 ***	2.6647 ***	2.3000 ***	2.3323 ***	−1.1676 **	−1.1762 **	−1.5308 ***	−1.5260 ***
	(4.97)	(4.99)	(4.23)	(4.30)	(−2.45)	(−2.47)	(−3.18)	(−3.17)
Year	yes	yes	yes	yes	yes	yes	yes	yes
Industry	yes	yes	yes	yes	yes	yes	yes	yes
Observations	1471	1471	1471	1471	1444	1444	1444	1444
Adjusted R^2^	0.3129	0.3161	0.3078	0.3092	0.2651	0.2651	0.2679	0.2682

Note: t statistics are in parentheses; ***, **, * represent the significance at the level of 1%, 5%, 10% respectively.

**Table 11:** Different CSR engagements in different periods.

Variable	Innovation							
	Before 2014				After 2014			
	(1)	(2)	(3)	(4)	(5)	(6)	(7)	(8)
CSR_ST	0.0218 ***	0.0212 ***			0.0120 ***	0.0116 ***		
	(9.45)	(9.16)			(4.73)	(4.60)		
CSR_SH			0.0902 ***	0.0899 ***			0.0739 ***	0.0738 ***
			(7.64)	(7.65)			(7.34)	(7.32)
FIN	−1.7562 ***	−1.5680 **	−1.7870 ***	−1.7790 ***	−1.3940 **	−1.7516 ***	−1.4408 ***	−1.4476 ***
	(−2.73)	(−2.51)	(−2.79)	(−2.78)	(−2.43)	(−2.94)	(−2.61)	(−2.61)
CSR_ST*FIN		−0.0636				−0.0817 **		
		(−1.62)				(−2.50)		
CSR_SH*FIN				−0.0525				−0.0902
				(−0.44)				(−1.04)
Leverage	0.4277 *	0.4185 *	0.6845 ***	0.6885 ***	0.7203 ***	0.7010 ***	0.8839 ***	0.8885 ***
	(1.69)	(1.65)	(2.76)	(2.78)	(3.00)	(2.91)	(3.61)	(3.64)
Age	−0.0179	−0.0190	−0.0159	−0.0158	−0.0033	−0.0053	−0.0013	−0.0017
	(−1.46)	(−1.54)	(−1.25)	(−1.24)	(−0.27)	(−0.43)	(−0.11)	(−0.15)
Asset turnover	0.4163 ***	0.4199 ***	0.3802 ***	0.3787 ***	0.3679 ***	0.3633 ***	0.3290 ***	0.3217 ***
	(3.87)	(3.91)	(3.42)	(3.40)	(3.57)	(3.51)	(3.24)	(3.15)
Tobin Q	−0.1355 ***	−0.1326 ***	−0.1065 ***	−0.1058 ***	−0.2321 ***	−0.2360 ***	−0.1885 ***	−0.1934 ***
	(−3.58)	(−3.48)	(−2.85)	(−2.82)	(−8.11)	(−8.30)	(−6.61)	(−6.74)
Liquidity	−0.9533 ***	−0.9586 ***	−1.2810 ***	−1.2855 ***	−0.8230 ***	−0.7907 ***	−0.8221 ***	−0.8059 ***
	(−3.54)	(−3.55)	(−4.72)	(−4.74)	(−3.01)	(−2.90)	(−3.02)	(−2.96)
Sales	−0.1154	−0.1127	−0.1809*	−0.1822*	−0.0672	−0.0600	−0.0245	−0.0254
	(−1.28)	(−1.25)	(−1.94)	(−1.95)	(−0.79)	(−0.70)	(−0.29)	(−0.30)
ROA	3.5344 ***	3.5115 ***	−0.9389	−0.9594	3.0883 ***	3.0312 ***	−2.8656 ***	−2.8653 ***
	(4.21)	(4.17)	(−0.86)	(−0.87)	(4.25)	(4.16)	(−2.75)	(−2.75)
Independ	−0.3868	−0.2967	−0.2744	−0.2442	−0.6818	−0.6368	−0.4920	−0.4961
	(−0.44)	(−0.33)	(−0.31)	(−0.28)	(−0.73)	(−0.68)	(−0.53)	(−0.53)
Hold	−0.9014 **	−0.9149 **	−0.8632 **	−0.8599 **	−0.2594	−0.3002	−0.4740	−0.4783
	(−2.48)	(−2.52)	(−2.33)	(−2.32)	(−0.65)	(−0.75)	(−1.21)	(−1.22)
Constant	2.1708 ***	2.1464 ***	1.4281 **	1.4128 **	2.1711 ***	2.2040 ***	1.4830 ***	1.4985 ***
	(4.21)	(4.17)	(2.54)	(2.51)	(4.66)	(4.74)	(3.13)	(3.16)
Year	yes	yes	yes	yes	yes	yes	yes	yes
Industry	yes	yes	yes	yes	yes	yes	yes	yes
Observations	1261	1261	1261	1261	1654	1654	1654	1654
Adjusted R^2^	0.2611	0.2627	0.2425	0.2426	0.2318	0.2342	0.2449	0.2454

Note: t statistics are in parentheses; ***, **, * represent the significance at the level of 1%, 5%, 10% respectively.

**Table 12:** Regression results of robustness tests.

Variable	Innovation			
	(1)	(2)	(3)	(4)
CSR	0.0130 ***		0.0131 ***	0.0131 ***
	(5.00)		(5.01)	(5.00)
FIN		−2.8217 ***	−2.8145 ***	−2.9114 ***
		(−4.17)	(−4.17)	(−4.22)
CSR*FIN				−0.0616 *
				(−1.66)
ROA	1.9221 **	3.6366 ***	2.0547 **	2.0287 **
	(2.02)	(3.98)	(2.15)	(2.12)
Leverage	0.8567 ***	0.6654 **	0.7522 **	0.7507 **
	(2.92)	(2.23)	(2.52)	(2.53)
Age	0.0045	0.0062	0.0076	0.0059
	(0.31)	(0.42)	(0.51)	(0.40)
Asset turnover	0.4771 ***	0.4145 ***	0.4239 ***	0.4133 ***
	(3.33)	(2.90)	(2.99)	(2.91)
Tobin Q	−0.2287 ***	−0.2492 ***	−0.2233 ***	−0.2252 ***
	(−6.41)	(−7.08)	(−6.17)	(−6.25)
Liquidity	−1.2584 ***	−1.3710 ***	−1.2711 ***	−1.2456 ***
	(−3.90)	(−4.28)	(−3.94)	(−3.86)
Sales	−0.1972 **	−0.2165 **	−0.1962 **	−0.1931 *
	(−2.02)	(−2.20)	(−1.99)	(−1.96)
Independ	−2.8999 ***	−2.8675 ***	−2.8636 ***	−2.7899 ***
	(−3.14)	(−3.20)	(−3.11)	(−3.02)
Hold	−1.9645 ***	−1.9823 ***	−2.1201 ***	−2.1490 ***
	(−5.01)	(−5.04)	(−5.38)	(−5.44)
Constant	2.1647 ***	2.7642 ***	2.2722 ***	2.2762 ***
	(4.16)	(5.40)	(4.33)	(4.33)
Year	yes	yes	yes	yes
Industry	yes	yes	yes	yes
Observations	2887	2887	2887	2887
Wald $\chi^{2}$	370.93	341.31	388.37	388.80
Pseudo R^2^	0.1302	0.1273	0.1355	0.1366

Note: z statistics are in parentheses; ***, **, * represent the significance at the level of 1%, 5%, 10% respectively.

**Table 13:** Robustness tests of different CSR engagements.

Variable	Innovation			
	(1)	(2)	(3)	(4)
CSR_ST	0.0125 ***	0.0125 ***		
	(4.38)	(4.38)		
CSR_SH			0.0672 ***	0.0672 ***
			(5.42)	(5.41)
FIN	−2.8095 ***	−2.9613 ***	−2.8401 ***	−2.8379 ***
	(−4.14)	(−4.24)	(−4.26)	(−4.26)
CSR_ST*FIN		−0.0925 **		
		(−2.16)		
CSR_SH*FIN				0.0429
				(0.39)
Leverage	0.7126 **	0.6975 **	0.8632 ***	0.8579 ***
	(2.39)	(2.34)	(2.90)	(2.87)
Age	0.0070	0.0048	0.0085	0.0087
	(0.48)	(0.33)	(0.56)	(0.58)
Asset turnover	0.4243 ***	0.4167 ***	0.4067 ***	0.4110 ***
	(3.00)	(2.94)	(2.82)	(2.85)
Tobin Q	−0.2336 ***	−0.2348 ***	−0.1975 ***	−0.1968 ***
	(−6.49)	(−6.54)	(−5.41)	(−5.38)
Liquidity	−1.2840 ***	−1.2527 ***	−1.3273 ***	−1.3314 ***
	(−3.98)	(−3.89)	(−4.11)	(−4.12)
Sales	−0.2015 **	−0.1952 **	−0.1927 *	−0.1920 *
	(−2.04)	(−1.98)	(−1.95)	(−1.94)
ROA	3.0653 ***	3.0248 ***	−1.4852	−1.4816
	(3.34)	(3.30)	(−1.17)	(−1.16)
Independ	−2.8983 ***	−2.8083 ***	−2.7070 ***	−2.7159 ***
	(−3.16)	(−3.06)	(−2.93)	(−2.97)
Hold	−2.0673 ***	−2.1128 ***	−2.2348 ***	−2.2352 ***
	(−5.25)	(−5.35)	(−5.63)	(−5.62)
Constant	2.4592 ***	2.4698 ***	1.8853 ***	1.8872 ***
	(4.73)	(4.47)	(3.48)	(3.48)
Year	yes	yes	yes	yes
Industry	yes	yes	yes	yes
Observations	2915	2915	2915	2915
Wald chi2	378.36	377.25	382.77	382.15
Pseudo R2	0.1335	0.1352	0.1367	0.1367

Note: z statistics are in parentheses; ***, **, * represent the significance at the level of 1%, 5%, 10% respectively.
